# The role of microenvironment in stem cell-based regeneration of intervertebral disc

**DOI:** 10.3389/fbioe.2022.968862

**Published:** 2022-08-09

**Authors:** Genglei Chu, Weidong Zhang, Feng Han, Kexin Li, Chengyuan Liu, Qiang Wei, Huan Wang, Yijie Liu, Fengxuan Han, Bin Li

**Affiliations:** ^1^ Orthopaedic Institute, Department of Orthopaedic Surgery, The First Affiliated Hospital, Suzhou Medical College, Soochow University, Suzhou, China; ^2^ Department of Orthopaedic Surgery, Affiliated Hospital of Nantong University, Nantong, China; ^3^ Collaborative Innovation Center of Hematology, Suzhou Medical College, Soochow University, Suzhou, China

**Keywords:** intervertebral disc, microenvironment, stem cell, tissue regeneration, regenerative medicine

## Abstract

Regenerative medicine for intervertebral disc (IVD) disease, by utilizing chondrocytes, IVD cells, and stem cells, has progressed to clinical trials in the treatment of back pain, and has been studied in various animal models of disc degeneration in the past decade. Stem cells exist in their natural microenvironment, which provides vital dynamic physical and chemical signals for their survival, proliferation and function. Long-term survival, function and fate of mesenchymal stem cells (MSCs) depend on the microenvironment in which they are transplanted. However, the transplanted MSCs and the endogenous disc cells were influenced by the complicated microenvironment in the degenerating disc with the changes of biochemical and biophysical components. It is important to understand how the MSCs and endogenous disc cells survive and thrive in the harsh microenvironment of the degenerative disc. Furthermore, materials containing stem cells and their natural microenvironment have good clinical effects. However, the implantation of tissue engineering IVD (TE-IVD) cannot provide a complete and dynamic microenvironment for MSCs. IVD graft substitutes may need further improvement to provide the best engineered MSC microenvironment. Additionally, the IVD progenitor cells inside the stem cell niches have been regarded as popular graft cells for IVD regeneration. However, it is still unclear whether actual IVD progenitor cells exist in degenerative spinal conditions. Therefore, the purpose of this review is fourfold: to discuss the presence of endogenous stem cells; to review and summarize the effects of the microenvironment in biological characteristics of MSC, especially those from IVD; to explore the feasibility and prospects of IVD graft substitutes and to elaborate state of the art in the use of MSC transplantation for IVD degeneration *in vivo* as well as their clinical application.

## 1 Introduction

The intervertebral disc (IVD) is the fibrocartilage connection of the adjacent vertebrae, like an elastic cushion, which can encounter the vibration of the spine from external forces and increase the amplitude of the spinal motion. As the senescence or other injury elements caused by multiple stresses occur and progressively develop, the degeneration of the IVD may happen, eventually leading to low back pain, disc herniation, etc., which impose a tremendous burden on global health. The IVD is avascular and can barely not repair itself (Chen et al., 2022). Currently, the molecular and pathogenesis mechanism of the degenerative disc disease is not totally understood. Surgery such as interbody fusion could relieve pain, but it restricted by the degeneration of the adjacent disc postoperation, so the present treatment is mainly physical therapies, anti-inflammatory medications and analgesic, all of which can not reverse the pathophysiological function of the degenerative IVD (Wu et al., 2020). Therefore, developing a new treatment to facilitate IVD regeneration is necessary.

Stem cells have multipotential to differentiate into many types of cells, which is a promising therapy and has already been applied to treating many diseases. Nowadays, many researchers have been studying on the stem cell-based treatment for IVD regeneration, such as mesenchymal stem cells (MSCs) (Yang et al., 2010; Sakai and Andersson, 2015). In recent years, more and more researchers have been focusing on the endogenous multipotent cells in the IVD. They reside in the IVD stem cell niche quiescently and can differentiate into chondrogenic cells, orthogenic cells and adipogenic cells after injuries or degeneration. However, when the IVD degeneration is ongoing, the disturbed catabolic-anabolic balance and the increasingly harsh microenvironment in the IVD might be unfavourable to progenitor cell activity (Chen et al., 2022). All above is a big challenge for stem cell-based treatments. Moreover, the properties of the IVD graft substitutes, like viscoelasticity, elasticity and microstructure, can determine the fate of stem cells (Chu et al., 2018; Gan et al., 2022). Hence, it is critical to figure out the effects of the materials-related microenvironment on the stem cell for IVD regeneration.

In this review, firstly, we will introduce the native IVD progenitors and the microenvironment changes in the progress of IVD degeneration. And then, the influence of the IVD microenvironment and IVD graft substitutes on the stem cells would be elucidated. At last, we will discuss the clinical applications and future perspectives based on the existing knowledge. We hope that this review can offer new insight for the repair and regeneration of IVD.

## 2 Tissue-specific progenitor cells in the IVD

**FIGURE 1:**
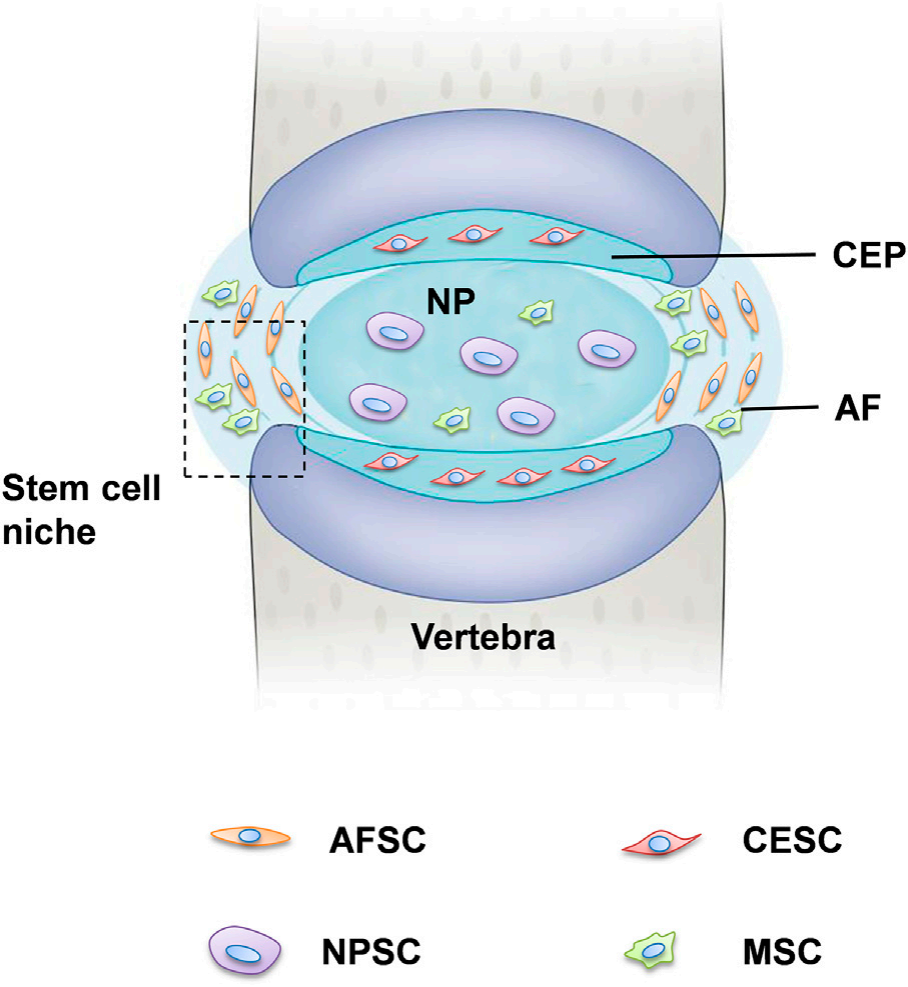
Tissue-specific progenitor cells and the location of stem cell niche. The IVD is the fibrocartilaginous part of a three three-component construct that consists of the nucleus pulposus (NP) in the center and annulus fibrosus (AF) in the surrounding, both of which are connected by cartilaginous endplates (CE). Progenitor cells have been found in all three constructs, respectively, referred to NPSCs, AFSCs, and CESCs. The perichondrium region adjacent to the epiphyseal plate and the AF border to the ligament zone has been suggested to be the IVD stem cell niche.

Stem cells usually stay quiescent in a region of the IVD, so-called stem cell niches, where they reside. Studies have suggested that IVD stem cell niches might exist in the outer zone of the AF as well as the perichondrium region adjacent to the epiphyseal plate (Figure 1) (Huang et al., 2011). Considering the ratio between the disc volume and the IVD cell number, it is reasonable to suppose the IVD as acellular, so the quantity of the progenitor cells is very small. Stem or progenitor cells have been proven to exist in the IVD in many species with the following supporting reasons. First, despite the avascular and aneural in IVD, the blood vessel and nerve growth, calcification and fibrocartilage-like tissue were found in the degenerated disc (Rutges et al., 2010). It is speculated that these pathological tissues might have derived from IVD progenitor and resident stem cells. Secondly, the application of autologous disc cells was beneficial for IVD regeneration. A number of these cells maintained self-renewal potential and multipotent differentiation, activating these progenitor cells could be a valuable strategy for IVD regeneration (Oehme et al., 2015; Hu et al., 2018).

Recently, tissue-specific progenitor cells have been identified in the IVD, and these cells could be targeted to promote intrinsic repair, suggested that IVD progenitor cells might have multiple origins. The tissue-specific progenitor cells are a form of three types of stem-like cells: annulus fibrosus-derived stem cells (AFSCs), nucleus pulposus-derived stem cells (NPSCs), and cartilage endplate-derived stem cells (CESCs) (Liang et al., 2017). Despite the different morphology in the structural organization of different anatomy of IVD, AFSCs, NPSCs and CESCs exhibited common multi-differentiation characteristics, proliferative and immunophenotype during cell culture (Blanco et al., 2010). Researchers have identified specific markers of IVD progenitor cells that are compatible with MSCs, such as CD105+, CD90^+^, CD73^+^, CD79^−^, CD34^−^, CD19^−^, CD14^−^, and HLA-DR (De Luca et al., 2020). Another study explored the multipotency of progenitor cells derived from NP cells, they found that NPSCs expressed stemness marker genes such as Nanog, CD133, OCT3/4, Sox2 and Nestin (Sakai et al., 2012; Huang S et al., 2013). Followed by: The current characterization and related study findings of IVD progenitor cell characteristics are described in Table 1. NPSCs are capable of differentiating into multiple neural cell types including neuron, oligodendrocyte and astroglial specific precursor cells *in vivo*, this provides a promising source of seeded cells for neural repair. Yao et al. identified that CESCs exist in the human degenerated CEP and are similar to bone-marrow mesenchymal stem cells (BMSCs) among the immunophenotype, proliferation rate, cell cycle, and stem cell gene expression (Yao et al., 2016). In our previous study, we separated and characterized a subpopulation of cells that possessed self-renewal, osteogenesis, and adipogenesis potential from rabbit AF tissue, and AFSCs exhibited several stem marker genes similar to that of MSCs (Liu et al., 2014). This suggests the AFSCs may be considered an ideal seeded cell for treating degenerative IVD. In general, IVD progenitor cells seem to meet the requirements for definition as MSCs, having multilineage potential expressing MSC markers, and being plastic adherent (Gan et al., 2021). However, further investigation of the *in vivo* characteristics is needed to fully address the cell localization, differentiation and functional role in the maintenance of IVD homeostasis (Kim et al., 2003).

**TABLE 1:** Surface markers of IVD progenitor cells.

Species	Cell type	Surface markers	Ref
Human	CESC	CD14^−^, CD19^−^, CD34^−^, CD45^−^, HLA-DR-, CD44^+^, CD73^+^, CD90^+^, CD105+, CD133+, CD166+, Stro-1+	Liu et al. (2011)
Human	CESC	HLA-DR-, CD14^−^, CD19^−^, CD34^−^, CD45^−^, CD73^+^, CD90^+^, CD105+	Yuan et al. (2018)
Human	AFSC, NPSC	CD34^−^, CD49a+, CD63^+^, CD73^+^, CD90^+^, CD105+, CD166+, p75 NTR+, CD133/1+	Risbud et al. (2007)
Human	IVDSC	CD90^+^, CD105+, Stro-1+	Brisby et al. (2013)
Rhesus macaque	NPSC	CD90^−^, CD45^−^, CD44^+^, CD90^+^, CD146+, CD166+, HLA-DR+	Huang et al. (2013a)
Human	NPSC	CD29^−^, CD45^−^, CD24^+^, CD73^+^, CD90^+^, CD105+	Qi et al. (2019)
Rat	NPSC	CD34^−^, CD45^−^, CD44^+^, CD90^+^, CD105+	Tao et al. (2015)
Human	NPSC	CD29^−^, CD44^−^, CD73^−^, CD90^−^, CD105-, CD29^+^, CD44^+^, CD73^+^, CD90^+^, CD105+	Shen et al. (2015)
Human	NPSC	CD34^−^, CD45^−^, CD73^+^, CD90^+^, CD105+	Lama et al. (2018)
Human	NPSC	HLA-DR-, CD34^−^, CD45^−^, CD73^+^, CD90^+^, CD105+	Liu et al. (2017)

## 3 The degenerated IVD microenvironment

**FIGURE 2:**
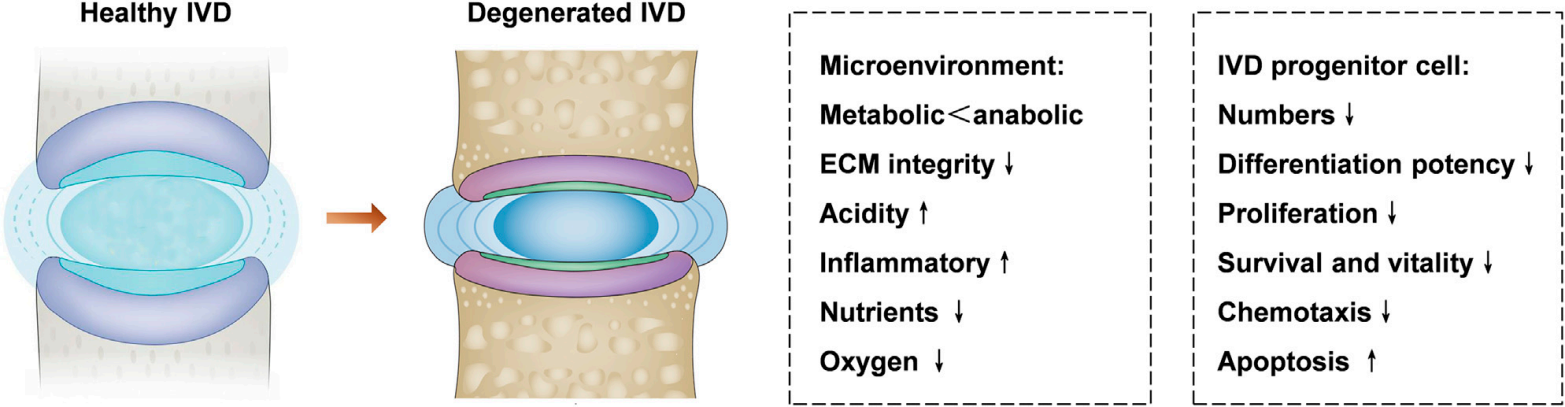
IVD microenvironment changes and IVD progenitor cells in healthy and degenerated IVD. The microenvironment of the degenerated IVD is characterized by metabolic disorders, acidic, inflammatory, poor nutrient supply, and low oxygen level, which offer a hostile microenvironment for IVD progenitor cells. Degenerative disc disease can lead to decreased differentiation potency, survival, chemotaxis, and proliferation of the IVD progenitor cells, the hostile microenvironment in the IVD might be unfavorable to the activity of progenitor cell.

To date, the pathophysiology of the degenerative disc disease is not completely clear, but it is accepted that aging, smoking, mechanical overload, obesity, and diabetes contribution to disc degeneration (Wang et al., 2017; Zhang Y. et al., 2021). IVD stem cells are involved in cell replenishment during wound healing and tissue regeneration. However, it is still ambiguous how IVD stem cells are exhausted during degeneration and aging (Gan et al., 2021). One possibility is that the increasingly harsh microenvironment and metabolic imbalance in the IVD might cause the progressive reduction of the number and the function of the stem cells (Figure 2). Another possibility is that after multiple rounds of differentiation and proliferation, the self-renewal ability of IVD stem cells becomes exhausted in response to degeneration-related or age-related damage. The possible reason includes nutrition deprivation, hypoxia, less reactive oxygen species, inflammatory factors, increased extracellular matrix (ECM) stiffness, and cyclic tension (Li et al., 2014; Zhang et al., 2020b; Zehra et al., 2022).

The calcification of the endplate happens as the IVD degeneration, which seriously influences the nourishment and oxygen supply of the IVD, would cause loss of NP ECM, NP cell death, and the collapse of IVD, leading to decreased height, eventually accelerating the progress of the IVD degeneration (Xu et al., 2021). Hypoxia can not only induce apoptosis and inhibit proliferation but also promote the IVD progenitor cells differentiate to chondrogenic cells (Huang S. et al., 2013). Yao et al. reported that the CESCs derived from degenerated IVDs had impaired osteogenic potential after the induction of HIF-1α or in hypoxic conditions (Yao et al., 2017a). In addition, the inflammatory cytokines (TNF-α, IL-1β, IL-8 and IL-6) could remodel the ECM metabolism from anabolism to catabolism, leading to an imbalance ECM and aggravating the pain response of IVD. The NPSCs with neurogenic potential may also contribute to innervation under inflammatory conditions, thus leading to DDD (Navone et al., 2012).

Substrate elasticity and stiffness occur as a result of the biomechanical changes that regulate the differentiation of stem cells. Cells can sense the interactions with ECM via integrin mediation. When the matrix stiffness microenvironment of the IVD changes, stem cells make corresponding alterations (Zhang W. et al., 2020). For example, NPSCs exhibit reduced chondrogenic gene expression but promoted osteogenic gene expression cultured in a stiff synthetic hydrogel matrix (Navaro et al., 2015). In our previous study, we investigate the effects of fiber size and stiffness of scaffolds on the differentiation of AFSC. We found that the fiber size and the stiffness of scaffolds play critical roles in regulating the microenvironment for stem cells (Chu G. L. et al., 2019). In addition, mechanical loading could induce ECM synthesis, thus regulating the fate of stem cells. It is proved that applied forces, such as compression, hydrostatic, and tension, could significantly affect the lineage specification and maintenance of AF progenitor cells (Stemper et al., 2014; Rasoulian et al., 2021). Furthermore, continuous cyclic tensile strain can induce apoptosis and osteogenic gene expression in human CESCs (Zuo et al., 2019).

Acidic conditions could lead to reduced proliferation, ECM production and cell viability of stem cells in degenerated IVD. Owing to low nutrition and hypoxia in IVD, there is plenty of lactic acid generated and accumulated within IVD, this leads to the IVD microenvironment being slightly acidic (between 7.0 and 7.2). Consequently, the pH of the IVD microenvironment would be lower, and the situation for cells to survive would be rougher (Han et al., 2014). According to previous studies, the pH may decrease to 6.5 in the early stages of IVD degeneration. In more severe cases, it can drop to 5.6 (Liu et al., 2017).

After all, these results demonstrate that the components of the IVD microenvironment could influence the physiological activity of progenitor cells. Several studies have tried to inject MSCs into the degenerate IVD, but rapidly the cells could not be detectable, which shows the severe effects of the microenvironment of the degenerate IVD on cells (Borem et al., 2019; Wangler et al., 2019). IVD stem cells seem to be both negatively and positively influenced by mechanical loading and hypoxia, whereas their physiological activity may be compromised by ECM stiffness changes, proinflammatory signaling, and acidic conditions. If stem cells or progenitor cells are truly resident in the healthy or degenerated disc, they will offer a new application for the regeneration of IVD.

## 4 The role of microenvironment in the application of stem cells-based IVD regeneration

Although stem cell transplantation therapy offers hope for IVD regeneration, their clinical use is still hampered by the harsh microenvironment of hypoxia, nutrition deprivation, acidic conditions, excessive cyclic tension, high osmolarity, and inflammatory factors (Huang Y. C et al., 2013). Therefore, appropriate regulation of the microenvironment could be used to enhance the physiological function of IVD stem cells in the complicated microenvironment.

### 4.1 Hypoxia and low nutrition

The IVD is inherently avascular, and the transport of nutrients and excretion of metabolites in IVD tissues strictly depends on the diffusion from capillaries that originate from the CEP. The lack of blood circulation establishes a hypoxic microenvironment with the average physiological oxygen tension (6 ± 2%) in human and falls to 1% in the degenerated IVD consequently (Peck et al., 2021). The properties of stem cells within a normoxia (20% O_2_) microenvironment is quite different from their performance in the IVD microenvironment that is hypoxia (1–5% O_2_). According to a recent study, hypoxia is beneficial in maintaining better stemness compared with normoxia. BMSCs exhibited greater colony-forming units and proliferated faster through the downregulation of E2A-p21 by Hypoxia-inducible factor 1 (HIF-1) (Elabd et al., 2018). Besides, when adipose-derived stem cells (ADSCs) stay in hypoxic and low glucose conditions *ex vivo*, their cell viability and the synthesis of the ECM increase (Hwang et al., 2020). Yao et al. demonstrated that physiological hypoxia promotes the chondrogenic differentiation of the CESCs (Yao et al., 2017b). Likewise, de Vries et al. observed BMSCs could differentiate to acquire phenotypes similar to that of NP cells after exposure in 2% O_2_ and 10 ng/ml transforming growth factor β (TGF-β) (de Vries et al., 2016). Hypoxia is an essential regulator for BMSCs, however, when the oxygen tension is less than 1%, prolonged exposure with serum deprivation would lead to complete cell mortality (Wang et al., 2020). It is reported that HIF is a transcriptional factor that can initiate cellular signals that regulate enzymes dealing with hypoxia. HIF is stable regardless of the oxygen concentration and can regulate cell proliferation, extracellular matrix production, energy supply, cell autophagy, and apoptosis (Luo et al., 2021). The absence of HIF would promote the degeneration of the IVD, and small leucine-rich proteoglycans (SLRPs) can activate HIF then help IVD stem cells survive in the hypoxic microenvironment (Meng et al., 2018; Silagi et al., 2021). Agents that could promote the upregulation of SLRPs might help NPSCs survive in the hypoxic microenvironment (Merceron et al., 2014).

Besides oxygen tension, glucose is another source of energy that significantly affects the differentiation, proliferation and viability of IVD progenitor cells. The consequence of low glucose availability is a decreased viability of IVD cells (Huang et al., 2014; Mahmoud et al., 2020). Some researchers have demonstrated the effect of glucose concentration on regulating the chondrogenic and osteogenic differentiation potential of CESCs to affect the differentiation of CESCs, which may represent a target for CE degeneration therapy (Sun et al., 2019). Furthermore, to adapt to the changes in the microenvironment, IVD progenitors correspondingly change their metabolism by increasing matrix synthesis and anaerobic glycolysis in low glucose conditions (5 mM), which allows generating energy while producing less reactive oxygen species (ROS) and consuming less O_2_ (Yin et al., 2020).

### 4.2 pH

The glucose concentration and oxygen tension are easy to be kept accurately to simulate the microenvironment of healthy IVD, the biggest challenge for IVD progenitor cells to survive in the microenvironment should be the ECM acidity, ranging from 7.0 to 7.2 in a healthy disc, whereas in degenerated IVD is between 6.8 and 6.2 (Cai et al., 2019). It has shown that under IVD-like acidic pH, the viability of BMSCs was impaired due to apoptosis and secondary necrosis, and the proliferation was also inhibited. In addition, this descending tendency was amplified within the lower pH between 7.4 and 6.5. Compared with ADSCs, NPSCs demonstrated less inhibition of viability and proliferation, which showed better performance to adapt to the acidic pH in IVD (Li et al., 2012). Another study also claimed that the acidic microenvironment of the degenerated IVD can induce BMSCs apoptosis by activating Ca^2+^-permeable ASIC1a (Cai et al., 2019). Additional modifiers might be used to enhance the function of progenitor cells located in the stem cell niche. A study demonstrated that the diuretic amiloride could block acid-sensing ion channels of cells and might help NPSCs to survive in an acidic microenvironment (Han et al., 2014).

### 4.3 Mechanical stimulation and osmotic conditions

Mechanical stimulation is a natural constituent of IVD, numerous studies have demonstrated the critical roles of mechanical loadings in regulating the fate of progenitor cells, including compression, shear, torsion, flexion and hydrostatic pressure (Fernando et al., 2011; Tsai et al., 2014). The intradiscal pressure of the healthy IVDs between L4 and L5 is about 0.1–0.24 MPa in the supine position. When the back is flexed with a loading of 20 kg, the intradiscal pressure can increase to 2.0 MPa (Liang et al., 2018). Moreover, the complex mechanical loading makes microenvironmental osmotic condition change. In daily life activities, the IVD osmolarity varies from 430 mOsm/L to 496 mOsm/L. Their osmolarity changes by around 25% during each diurnal cycle. The main component to regulate the IVD osmotic pressure is negatively charged sulphated GAGs linked with aggrecan, which can intake the water into the IVD tissue. The IVD osmotic conditions is fluctuating all the time corresponding to many factors such as tissue hydration, mobile ions transportation and disc loading (Lyu et al., 2019). IVD osmolarity has different impacts on the stem cells owing to its variation. Zhang et al. demonstrated that hyperosmolarity inhibited ADSCs proliferation and viability, promoted the ADSCs differentiate to NP-like cells, activated Foxa1/2-Shh signaling and increased the expression of KDM4B and (Zhang et al., 2020c).

**FIGURE 3:**
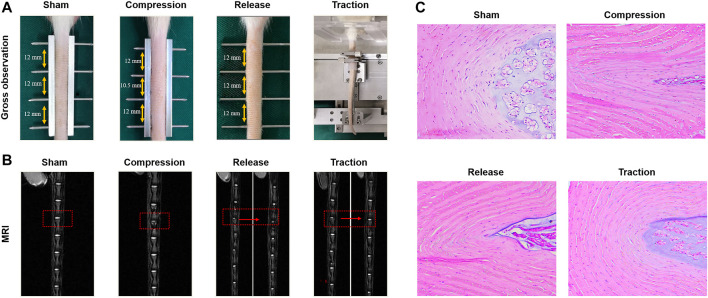
Effects of different modes of mechanical loading on the influence of IVD *in vivo*. **(A)** Gross observation of rat tail IVDs in the sham group, compression group, release group and traction group. **(B)** Magnetic resonance images (MRI) of IVDs from the groups as described above. **(C)** H&E staining of IVDs from the groups as described above. Reproduced with permission from Zhang et al. (Zhang W. et al., 2021).

External mechanical stimuli also influence the migration, inflammatory response, proliferation and ECM synthesis of stem cells, which depend on the loading level and mode. Our study demonstrated that AFSCs presented different behaviors when subject to cyclic tensile strain with the magnitudes of 2, 5, and 12%. The catabolic and inflammatory gene increased at high magnitude of cyclic tensile strain with 12% by activating Caveolin-1 mediated NF-κB and integrin β1 signaling pathways. However, cell proliferation, migration and anabolism were found to increase after exposure to moderate cyclic tensile strain with 5% magnitude (Zhang W. et al., 2021). Hence, AFSCs seem to be affected positively and negatively by mechanical stimulation, and physiological mechanical loading might play a therapeutic role in AF regeneration (Figure 3).

### 4.4 Proteinase and cytokines

The ECM is biochemical and structural support to the IVD stem cells. There are collagen type I, collagen type II, hyaluronic acid (HA) and other components in the ECM. ECM can retain water, arrange collagen tissue and provide viscoelastic properties (Penolazzi et al., 2020). The IVD degeneration is related to excessive matrix catabolism. Many cytokines and endogenous proteases increased during the degeneration progress, creating another complicated microenvironment for stem cell transplantation. The interaction between matrix metalloproteinases (MMPs) and tissue inhibitor of metalloproteinases (TIMPs) is biologically important for the remodeling of IVD tissue, which could impact many aspects of the cells, such as viability, proliferation, differentiation and apoptosis (Hingert et al., 2020). Therefore, maintaining the ECM metabolism of natural tissues is very important. A mechanotransducer-targeted drug that mediates ECM stiffness to maintain the phenotype of IVD progenitor cells can prevent IVD degeneration. A study reported that the upregulation of TIMPs could restore the balance between catabolic and anabolic environments, suggesting TIMPs might be a possible therapeutic target for promoting the IVD regeneration (Le Maitre et al., 2004).

Apart from the effects of MMPs and TIMPs on stem cells, interleukins and other pro-inflammatory cytokines in IVD also regulated the behavior of stem cells. First, Interleukins (IL-1α, IL-1β) and TNF-α, prostaglandin E2 (PGE2), chemokines, and IFNγ played an important role in inducing the cell apoptosis, autophagy, and senescence to upregulate the synthesis of ADAMTS (-1, -4, -5, -9, and -15) and MMPs (-1, -3, -7, -9, and -13), thus leading to ECM breakdown and the development of discogenic low back pain (Jacobsen et al., 2021; Kim et al., 2021). Furthermore, TNF-α has been demonstrated to inhibit the differentiation of adipocytes, as well as the adipogenesis of stem cells. Besides, TNF-α plays a critical role in ECM remodeling by reducing the expression of YAP/TAZ and consequently impairing the chondrocytogenesis of IVD progenitor cells (Ekram et al., 2021).

Conversely, many cytokines also indicate a more favorable outcome to treatment. It was shown that many interleukins exhibit chemotactic features, which might play an important role in stem cell recruitment into degenerated IVD (Zhao et al., 2020). Besides, IVD progenitor cells have been demonstrated to secrete cytokines and proteinase that support NP cell functionality and viability under stress. For instance, insulin-like growth factor 1 (IGF-1) and TGF-β have been described to reduce ECM degradation and inflammation of IVD tissue, while bone morphogenetic protein 7 (BMP-7) and IGF-1 protected NP cells against apoptosis (Yang et al., 2015). Moreover, many interleukins significantly upregulated the immunosuppressive ability of stem cells. It was shown that IL-6 regulated the stemness of stem cells and maintained the undifferentiated state and the proliferation of MSCs through ERK signaling pathway (Marimuthu and Pushpa Rani, 2021). Because of the complicated molecular network in the degenerative disc disease, the synergistic and antagonistic effects of proteinase and cytokines on stem cells required further investigation.

## 5 Role of IVD graft substitutes in stem cell microenvironment

An ideal graft for IVD regeneration should provide prolonged and instant mechanical properties, biochemical cues and adequate space for seeded stem cells to differentiate, proliferation, and produce ECM (Wei et al., 2021; Luo et al., 2022). Illustrating the interactive mechanisms between the components such as seeded stem cells, environmental factors, ECM components and biological factors in the IVD microenvironment makes therapeutic strategies more rational. In addition, the hostile degenerated IVD microenvironment will be considered when developing IVD substitutes. The IVD graft substitutes should mimic the native IVD tissue with good mechanical strength to withstand the multidirectional and complex mechanical stimulations. Considering the pathological changes of degenerative disc disease, they should also mimic the anisotropic biochemical property, viscoelasticity and topography of native IVD tissue, meeting biological and mechanical compatibilities are crucial for the longevity and efficacy of the IVD repair and regeneration.

### 5.1 Elasticity

Substrate elasticity and stiffness have regulatory in stem cell adhesion, migration, proliferation and differentiation, thus become an important designing factor in IVD tissue engineering. NPSCs cultured in a stiff synthetic hydrogel matrix showed reduced chondrogenic gene expression and increased osteogenic gene expression (See et al., 2012). In addition, the elastic modulus of the scaffolds was found to affect stem cell fate and direct their lineage specification significantly. Recently, in order to mimic the gradient stiffness close to that of native AF tissue, we fabricated a series of electrospun poly (ether carbonate urethane) urea membranes and evaluated the behavior of AFSCs cultured on it. The expression of phenotypic marker genes of inner AF zone decreased with the substrate stiffness, while the expression of phenotypic marker genes of outer AF zone showed an opposite trend (Zhu et al., 2016). Similarly, Wan et al. fabricated a biphasic substrate to structurally and elastically mimic the AF, the outer phase of the scaffold is demineralized bone matrix gelation (BMG), while the inner part of the scaffold is poly (polycaprolactone triol malate) (PPCLM). The biphasic structure attempt to recapitulate the distribution and mechanical properties of native AF, thus offers enhanced tensile stress and compressive strength than uniphasic scaffold, making it a promising candidate for AF repair and regeneration (Wan et al., 2008).

NP replacement has been explored in various stages of clinical development for the treatment of degenerative disc disease. A variety of synthetic polymeric materials and natural materials such as collagen, chitosan, agarose, and alginate have been studied for creating a successful engineered NP tissue replacement, and the equilibrium Young modulus (E_y_) of unconfined human NP was ∼5 kPa, and a percent relaxation of ∼65% (Yao et al., 2006). Lin et al. used photocross-linked alginate to encapsulate bovine NP cells, which promote the proliferation and maintain the viability of the seeded cells *in vitro* and *in vivo* (Lin et al., 2016).

After all, physical cues such as elasticity and stiffness, originated from cellular microenvironment, can direct stem cell differentiation and influence the physiological function of cultured stem cells. These findings will facilitate IVD bioengineering with mechanical functions approximate to the native IVD tissue for IVD regeneration.

### 5.2 Viscoelasticity

Besides the commonly known elasticity, a number of studies have demonstrated that viscoelasticity of matrix markedly affects cellular physiological functions, such as differentiation, proliferation and cell spreading (Lang et al., 2021; Jia et al., 2022). When cultured in hydrogel (17 kpa) with fast relaxation rate, MSCs preferentially differentiate toward osteoblast and produce rich mineralized collagen type I (Yue et al., 2019). In order to recapitulate the fibrillar architecture and viscoelasticity of the ECM, Lou et al. develop an interpenetrating network hydrogel system by using the crosslink of hyaluronic acid with collagen through dynamic covalent. They demonstrated that the faster relaxation of the interpenetrating network hydrogel system could improve the fiber remodeling, cell spreading, and hyaluronic acid formation of stem cells embedded in this system (Lou et al., 2018).

Degenerative disc disease is associated with alternations in the biochemical composition of the IVD tissue, such as increased collagen deposition, decreased proteoglycan concentration, and decreased water content, which would have great influence on mechanical function of IVD in shear and compression. Such changes may also affect the dynamic viscoelasticity of IVD tissue and thus alter the ability of IVD to dissipate energy under physiologic loading (Lee and Teo, 2004). In addition, IVD cells within tissues use their contractile machinery to probe and surveil the local microenvironment in both healthy and diseased states. By utilizing a puncture model in rabbit AF, Bonnevie et al. showed that the loss of residual strain in ruptured AF could lead to aberrant cellular changes to fibrotic phenotype and promote the short-term apoptosis of AF cells (Bonnevie et al., 2019). In addition, Eberhardsteiner et al. suggested that viscoelasticity of the AF ECM function is essential in resistance to bulk tissue failure by stress distribution between fibers (Eberhardsteiner et al., 2014). Therefore, viscoelasticity changes initiated the activation of aberrant mechanotransductive events with a loss of prestrain that targets cellular contractility and ultimately leads to disc degeneration.

### 5.3 Topography

Besides stiffness or viscoelasticity of the ECM, topographic factors including shape, size, and geometric arrangement are also considered critical biophysical cue that influences cell behaviors (Johnson et al., 2006; Peng et al., 2021). Bhattacharjee et al. fabricated scaffolds with fibers alignment that mimics the fibrous orientation of AF by using silk fibroin, the fibers offer the chondrogenic re-differentiation support and the cell/ECM alignment (Bhattacharjee and Ahearne, 2020). At both micro-and nano-scale, behaviors of stem cells can be significantly influenced by the size of topographical features. In a study using aligned nanofibrous scaffolds which resembled nonlinear dependence of modulus native AF. Research have demonstrated that the scaffolds guided the alignment of AF cells and formed ECM with considerable alignment (Nerurkar et al., 2011).

**FIGURE 4:**
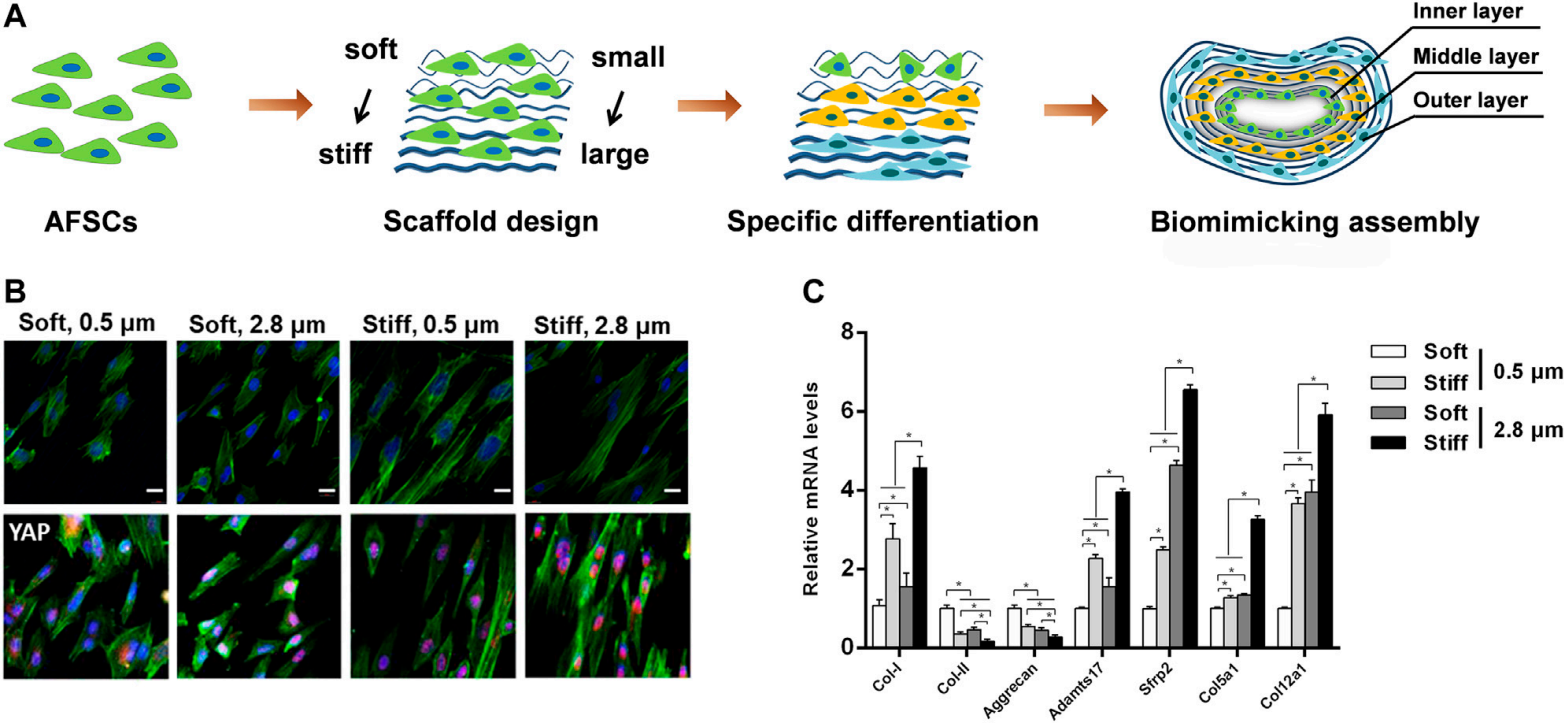
Combined effects of substrate topography and stiffness on the influence of AFSC differentiation. **(A)** Assembly of electrospun fibrous membranes with different fiber size and stiffness led to specifically differentiated AFSCs that mimic the hierarchical stratified structure of native AF tissue. Reproduced with permission from Zhou et al. (Zhou et al., 2021). **(B)** The morphology and YAP expression of AFSCs on scaffolds. **(C)** Expression of AF phenotypic marker genes cultured on electrospun fibrous membranes scaffolds for 7 days. Reproduced with permission from Chu et al. (Chu G. et al., 2019).

In order to engineer the structural anisotropy and key length scales of AF, aligned fibrous scaffolds with different fiber sizes are currently preferred for AF regeneration (Figure 4). Our group recently found that the fiber size of the grafts significantly affected the morphology and differentiation of stem cells cultured on it, AFSCs were able to differentiate into cell phenotypes similar to AF cells in various zones. Moreover, we found that microstructure features and mechanical property of firbous scaffolds exert a combine effect on AFSC differentiation, possibly through a Yes-associated protein (YAP) dependent mechanotransduction mechanism. (Chu G. et al., 2019; Chu et al., 2021). Above all, the topographical and geometric arrangement of graft could regulate physiological behavior of stem cells such as differentiation, matrix synthesis and apoptosis.

### 5.4 Chemical composition

In addition to providing topographic support and mechanical stimulus for cells, IVD grafts could also be loaded with bioactive agents (Guan et al., 2021; Chen and Jiang, 2022). In order to achieve antioxidant properties in NP, Cheng et al. fabricated an injectable thermosensitive hydrogel scaffold incorporated with ferulic acid as a controlled release system. After loading with the ferulic acid, the hydrogel scaffolds achieved excellent antioxidant properties (Cheng et al., 2013). Vadala et al. fabricated a bioactive electrospun PLLA scaffold with TGF-β1 for the repair and regeneration of damaged AF, the PLLA/TGF scaffold promoted cell proliferation as well as glycosaminoglycan and collagen production. The slow-releasing profile of TGF-β1 from the scaffold exhibited a sustained delivery that allows envisaging an application for AF repair and tissue engineering strategies (Vadala et al., 2012). Conventional technologies have constructed amounts of scaffolds with single bioactive factor, which have limitations in replicating the composition of native tissues or organs. Bao et al. reported an anti-bacterial PCL scaffolds incorporation of antimicrobial agents for AF tissue engineering, the combining scaffolds showed a sustained strong anti-bacterial and anti-fungal activity after 1 month (Bao et al., 2013). In a study using silk hydrogels recombined with human IGF and BMP-2 could induce the stem cells to differentiate into chondrogenic and osteogenic phenotypes. The silk hydrogels are finally used for the regeneration of EP cartilage-to-bone interface (Wang et al., 2009). Guillaume et al. developed a covalently cross-linked alginate hydrogel combined with continuous delivery of active compounds as FGF-2 and TGF-β3, the combined porous scaffolds exhibit successful ECM deposition to promote the regeneration of AF defects (Guillaume et al., 2014). Above all, substrates combined with bioactive factors attract increasing attention for IVD regeneration.

## 6 Clinical applications and future perspectives

There have been promising results in clinical trials for stem cell treatment of LBP. Therapies that have been verified helpful in preclinical trials are in different phases of being attempted in clinical trials to demonstrate their safety and efficacy (Urits et al., 2019; Binch et al., 2021). In a case of EuroDISC study, IVD cells were introduced to the discs after discectomy. No adverse events were found in clinical trials, and relief of pain was noted in the injection group compared to discectomy alone on a 2-years follow-up. Furthermore, patients who underwent discectomy and autologous disc chondrocyte transplantation were proved to have less adjacent segment disease than those who underwent discectomy alone (Yoshikawa et al., 2010). In another clinical trial, autologous MSCs were injected into the disc of 13 patients followed for 1 year as part of a pilot study. Results have shown the technique to be feasible and safe, while there was no disc height change in these patients, some benefits with pain relief occurred in most patients, and improved disc hydration was noted in 10/13 on MRI at 6 months (Orozco et al., 2011). Another study by Henriksson et al. traced the MSC injected into the discs of patients who subsequently undergoing spinal fusion surgery, the investigators were able to detect the labeled MSCs and found that the MSCs had the tendency of differentiating into chondrocyte-like cells (Henriksson et al., 2012). These measurements proved to compare favorably to disc replacement and spinal fusion while offering the benefit of being less invasive (Table 2). However, despite the encouraging clinical trials outcomes, there are many difficulties that remain prior to the cell-based therapies in clinical applications. The biggest obstacle was the harsh microenvironment such as hypoxia, nutrition deprivation, acidic conditions, excessive cyclic tension, high osmolarity, and inflammatory factors during the disc degeneration. The hostile microenvironment that may negatively impact cell-based and biological therapy in the progress of disc regeneration (Kong et al., 2020). According to the results of the clinical and experimental trials, stem cells could be effective to mildly degenerative disc, but not be sufficient to support the view that the stem cells will regenerate the advanced degenerative IVD (Du et al., 2021). In NP cells, HIF is stable in an oxygen-independent fashion, which enables NP cells to survive the harsh microenvironment. Future studies of cell-based therapy should focus on identifying the correct properties of microenvironment to adapt to the harsh environment that is present (Guerrero et al., 2021). Furthermore, the EP role as the nutrition transport regulator for disc regeneration and degeneration. The potential cross-talk between the cartilaginous EP and transplanted MSCs may open a new dimension to investigate the regenerative mechanisms.

**TABLE 2:** Clinical studies utilizing cell-based therapies to treat chronic low back pain.

Author, year	Clinical details	Cells transplanted	Results
Orozco et al. (2011)	10 patients with low back pain and evidence of DDD	Autologous MSCs	Clinical improvement in back pain, leg pain and disability. Disc height not recovered. Increased MRI T2 signal
Yoshikawa et al. (2010)	2 patients with back pain and sciatica, with radiological evidence of DDD and lumbar canal stenosis	Autologous marrow MSCs	Clinical improvement in both patients. Increased MRI T2 signal. Less instability
Henriksson et al. (2019)	4 patients undergo MSC injection and who later opted for spinal surgery	autologous MSCs	The labelled MSCs have differentiated into chondrocyte-like cells and were distributed at different parts of the IVD
Centeno et al. (2017)	33 patients with lower back pain and disc bulge	Autologous BM-MSCs	Mean 60% Clinical improvement in all patients. MRI showed a reduction in disc bulge. Patients with greater bulge reduction reported less pain
Kumar et al. (2017)	10 patients with low back pain due to DDD	Autologous AT-MSCs	No statistical difference between high and low dose arms. 1 Patient with improved Pfirmann grade. Greater than 50% (Oswestry Disability Index) ODI and (visual analog scale) VAS in 13 patients
Elabd et al. (2016)	5 patients with back pain and sciatica	Autologous hypoxic-cultured BM-MSCs	A positive association between clinical improvement and stem cell therapies. MRI showed reduced disc protrusion
Noriega et al. (2017)	24 patients with low back pain	Allogenic BM-MSCs	MRI showed improvement in MSC patients VS. disc degeneration in controls. Stem cell patients had significant ODI VAS and ODI VAS reductions at 3, 6, 12 months. MRI showed disc degeneration in controls VS. improvement in MSC patients
Meisel et al. (2007)	28 patients undergoing microdiscectomy with back pain	Autologous culture expanded disc derived chondrocytes	Patients receiving cell transplantation had reduced back pain at 2 years. Increased MRI T2 signal of treated and adjacent discs

## 7 Concluding remarks

Over the past decade, understanding the potential stem cell-based clinical application for IVD regeneration has dramatically improved. These advances have led to the development of new candidate therapies and, in some cases, clinical trials. So far, the use of stem cells for IVD repair and degeneration is still at the stage of pre-clinical and Phase 1 stage, mainly due to the low survival rate post-transplantation, the complicated microenvironment cells interact with and the detail functions for IVD regeneration are not fully understood. Among the harsh microenvironment posed to biological therapy for the degenerative disc disease are the chronic inflammation, reduced PH, excessive mechanical loading, and metabolic disorders. To overcome these challenges, it is necessary to figure out the influence of the microenvironment on the stem cell for IVD regeneration. Newly designed IVD graft substitutes may be able to simultaneously regulate cellular physiological function, and the controlled release of bioactive factors and therapeutic drugs while providing proper structural support, antibiosis, anti-inflammation. However, most strategies are still in the experimental stage, the relief of the symptoms do not mean that degeneration has stopped, as IVDs remain in a hostile microenvironment. Every therapeutic strategy has a deficiency. Future studies should pay more attention to making full use of biomaterials-mediated delivery of biomechanics or mechanical stimulation to stimulate stem cell differentiation and survival effectively. Therefore, an in-depth understanding of the microenvironment during degeneration and regeneration of IVD will help find a new way to restore the homeostatic microenvironment of IVD and ultimately achieve effective treatment for degenerative disc disease.

## References

[B1] Bao J. Lv W. Sun Y. Y. Deng Y. (2013). Electrospun antimicrobial microfibrous scaffold for annulus fibrosus tissue engineering. J. Mat. Sci. 48 (12), 4223–4232. 10.1007/s10853-013-7235-7

[B2] Bhattacharjee P. Ahearne M. (2020). Fabrication and biocompatibility of electroconductive silk fibroin/PEDOT: PSS composites for corneal epithelial regeneration. Polym. (Basel) 12 (12), 3028. 10.3390/polym12123028 PMC7766233 33348815

[B3] Binch A. L. A. Fitzgerald J. C. Growney E. A. Barry F. (2021). Cell-based strategies for IVD repair: Clinical progress and translational obstacles. Nat. Rev. Rheumatol. 17 (3), 158–175. 10.1038/s41584-020-00568-w 33526926

[B4] Blanco J. F. Graciani I. F. Sanchez-Guijo F. M. Muntion S. Hernandez-Campo P. Santamaria C. (2010). Isolation and characterization of mesenchymal stromal cells from human degenerated nucleus pulposus: Comparison with bone marrow mesenchymal stromal cells from the same subjects. Spine 35 (26), 2259–2265. 10.1097/BRS.0b013e3181cb8828 20622750

[B5] Bonnevie E. D. Gullbrand S. E. Ashinsky B. G. Tsinman T. K. Elliott D. M. Chao P. G. (2019). Aberrant mechanosensing in injured intervertebral discs as a result of boundary-constraint disruption and residual-strain loss. Nat. Biomed. Eng. 3 (12), 998–1008. 10.1038/s41551-019-0458-4 31611678 PMC6899202

[B6] Borem R. Madeline A. Bowman M. Gill S. Tokish J. Mercuri J. (2019). Differential effector response of amnion- and adipose-derived mesenchymal stem cells to inflammation; implications for intradiscal therapy. J. Orthop. Res. 37 (11), 2445–2456. 10.1002/jor.24412 31287173

[B7] Brisby H. Papadimitriou N. Brantsing C. Bergh P. Lindahl A. Barreto Henriksson H. (2013). The presence of local mesenchymal progenitor cells in human degenerated intervertebral discs and possibilities to influence these *in vitro*: a descriptive study in humans. Stem Cells Dev. 22 (5), 804–814. 10.1089/scd.2012.0179 23025667

[B8] Cai F. Hong X. Tang X. Liu N. C. Wang F. Zhu L. (2019). ASIC1a activation induces calcium-dependent apoptosis of BMSCs under conditions that mimic the acidic microenvironment of the degenerated intervertebral disc. Biosci. Rep. 39 (11), BSR20192708. 10.1042/BSR20192708 31696219 PMC6851507

[B9] Centeno C. Markle J. Dodson E. Stemper I. Williams C. J. Hyzy M. (2017). Treatment of lumbar degenerative disc disease-associated radicular pain with culture-expanded autologous mesenchymal stem cells: a pilot study on safety and efficacy. J. Transl. Med. 15, 197. ARTN 197. 10.1186/s12967-017-1300-y 28938891 PMC5610473

[B10] Chen D. Jiang X. (2022). Exosomes-derived miR-125-5p from cartilage endplate stem cells regulates autophagy and ECM metabolism in nucleus pulposus by targeting SUV38H1. Exp. Cell Res. 414 (1), 113066. 10.1016/j.yexcr.2022.113066 35231441

[B11] Chen S. Chen M. Wu X. Lin S. Tao C. Cao H. (2022). Global, regional and national burden of low back pain 1990-2019: a systematic analysis of the global burden of disease study 2019. J. Orthop. Transl. 32, 49–58. 10.1016/j.jot.2021.07.005 PMC8639804 34934626

[B12] Cheng Y. H. Yang S. H. Liu C. C. Gefen A. Lin F. H. (2013). Thermosensitive hydrogel made of ferulic acid-gelatin and chitosan glycerophosphate. Carbohydr. Polym. 92 (2), 1512–1519. 10.1016/j.carbpol.2012.10.074 23399183

[B13] Chu G. L. Yuan Z. Q. Zhu C. H. Zhou P. H. Wang H. Zhang W. D. (2019b). Substrate stiffness- and topography-dependent differentiation of annulus fibrosus-derived stem cells is regulated by Yes-associated protein. Acta Biomater. 92, 254–264. 10.1016/j.actbio.2019.05.013 31078765

[B14] Chu G. Shi C. Wang H. Zhang W. Yang H. Li B. (2018). Strategies for annulus fibrosus regeneration: From biological therapies to tissue engineering. Front. Bioeng. Biotechnol. 6, 90. 10.3389/fbioe.2018.00090 30042942 PMC6048238

[B15] Chu G. Yuan Z. Zhu C. Zhou P. Wang H. Zhang W. (2019a). Substrate stiffness- and topography-dependent differentiation of annulus fibrosus-derived stem cells is regulated by Yes-associated protein. Acta Biomater. 92, 254–264. 10.1016/j.actbio.2019.05.013 31078765

[B16] Chu G. Zhang W. Zhou P. Yuan Z. Zhu C. Wang H. (2021). Substrate topography regulates differentiation of annulus fibrosus-derived stem cells via CAV1-YAP-mediated mechanotransduction. ACS Biomater. Sci. Eng. 7 (3), 862–871. 10.1021/acsbiomaterials.9b01823 33715372

[B17] De Luca P. Castagnetta M. de Girolamo L. Coco S. Malacarne M. Ragni E. (2020). Intervertebral disc and endplate cell characterisation highlights annulus fibrosus cells as the most promising for tissue-specific disc degeneration therapy. Eur. Cell. Mat. 39, 156–170. 10.22203/eCM.v039a10 32125689

[B18] de Vries S. A. van Doeselaar M. Meij B. P. Tryfonidou M. A. Ito K. (2016). The stimulatory effect of notochordal cell-conditioned medium in a nucleus pulposus explant culture. Tissue Eng. Part A 22 (1-2), 103–110. 10.1089/ten.TEA.2015.0121 26421447

[B19] Du Y. X. Wang Z. K. Wu Y. M. Liu C. Y. Zhang L. L. (2021). Intervertebral disc stem/progenitor cells: a promising "seed" for intervertebral disc regeneration. Stem Cells Int. 2021, 1–12. Artn 2130727. 10.1155/2021/2130727 PMC8342144 34367292

[B20] Eberhardsteiner L. Hellmich C. Scheiner S. (2014). Layered water in crystal interfaces as source for bone viscoelasticity: Arguments from a multiscale approach. Comput. Methods Biomech. Biomed. Engin. 17 (1), 48–63. 10.1080/10255842.2012.670227 PMC3877913 22563708

[B21] Ekram S. Khalid S. Bashir I. Salim A. Khan I. (2021). Human umbilical cord-derived mesenchymal stem cells and their chondroprogenitor derivatives reduced pain and inflammation signaling and promote regeneration in a rat intervertebral disc degeneration model. Mol. Cell. Biochem. 476 (8), 3191–3205. 10.1007/s11010-021-04155-9 33864569

[B22] Elabd C. Centeno C. J. Schultz J. R. Lutz G. Ichim T. Silva F. J. (2016). Intra-discal injection of autologous, hypoxic cultured bone marrow-derived mesenchymal stem cells in five patients with chronic lower back pain: a long-term safety and feasibility study. J. Transl. Med. 14, 253. 10.1186/s12967-016-1015-5 27585696 PMC5009698

[B23] Elabd C. Ichim T. E. Miller K. Anneling A. Grinstein V. Vargas V. (2018). Comparing atmospheric and hypoxic cultured mesenchymal stem cell transcriptome: Implication for stem cell therapies targeting intervertebral discs. J. Transl. Med. 16 (1), 222. 10.1186/s12967-018-1601-9 30097061 PMC6086019

[B24] Fernando H. N. Czamanski J. Yuan T. Y. Gu W. Salahadin A. Huang C. Y. (2011). Mechanical loading affects the energy metabolism of intervertebral disc cells. J. Orthop. Res. 29 (11), 1634–1641. 10.1002/jor.21430 21484859 PMC3137745

[B25] Gan D. Jiang Y. Hu Y. Wang X. Wang Q. Wang K. (2022). Mussel-inspired extracellular matrix-mimicking hydrogel scaffold with high cell affinity and immunomodulation ability for growth factor-free cartilage regeneration. J. Orthop. Transl. 33, 120–131. 10.1016/j.jot.2022.02.006 PMC8914478 35330942

[B26] Gan Y. He J. Zhu J. Xu Z. Wang Z. Yan J. (2021). Spatially defined single-cell transcriptional profiling characterizes diverse chondrocyte subtypes and nucleus pulposus progenitors in human intervertebral discs. Bone Res. 9 (1), 37. 10.1038/s41413-021-00163-z 34400611 PMC8368097

[B27] Guan Y. Sun C. Zou F. Wang H. Lu F. Song J. (2021). Carbohydrate sulfotransferase 3 (CHST3) overexpression promotes cartilage endplate-derived stem cells (CESCs) to regulate molecular mechanisms related to repair of intervertebral disc degeneration by rat nucleus pulposus. J. Cell. Mol. Med., jcmm.16440. 10.1111/jcmm.16440 PMC8256370 33993645

[B28] Guerrero J. Hackel S. Croft A. S. Hoppe S. Albers C. E. Gantenbein B. (2021). The nucleus pulposus microenvironment in the intervertebral disc: The fountain of youth? Eur. Cell. Mat. 41, 707–738. 10.22203/eCM.v041a46 34128534

[B29] Guillaume O. Daly A. Lennon K. Gansau J. Buckley S. F. Buckley C. T. (2014). Shape-memory porous alginate scaffolds for regeneration of the annulus fibrosus: Effect of TGF-β3 supplementation and oxygen culture conditions. Acta Biomater. 10 (5), 1985–1995. 10.1016/j.actbio.2013.12.037 24380722

[B30] Han B. Wang H. C. Li H. Tao Y. Q. Liang C. Z. Li F. C. (2014). Nucleus pulposus mesenchymal stem cells in acidic conditions mimicking degenerative intervertebral discs give better performance than adipose tissue-derived mesenchymal stem cells. Cells Tissues Organs 199 (5-6), 342–352. 10.1159/000369452 25661884

[B31] Henriksson H. B. Hagman M. Horn M. Lindahl A. Brisby H. (2012). Investigation of different cell types and gel carriers for cell-based intervertebral disc therapy, *in vitro* and *in vivo* studies. J. Tissue Eng. Regen. Med. 6 (9), 738–747. 10.1002/term.480 22072598

[B32] Henriksson H. B. Papadimitriou N. Hingert D. Baranto A. Lindahl A. Brisby H. (2019). The traceability of mesenchymal stromal cells after injection into degenerated discs in patients with low back pain. Stem Cells Dev. 28 (17), 1203–1211. 10.1089/scd.2019.0074 31237488

[B33] Hingert D. Ekstrom K. Aldridge J. Crescitelli R. Brisby H. (2020). Extracellular vesicles from human mesenchymal stem cells expedite chondrogenesis in 3D human degenerative disc cell cultures. Stem Cell Res. Ther. 11 (1), 323. 10.1186/s13287-020-01832-2 32727623 PMC7391655

[B34] Hu B. He R. Ma K. Wang Z. Cui M. Hu H. (2018). Intervertebral disc-derived stem/progenitor cells as a promising cell source for intervertebral disc regeneration. Stem Cells Int. 2018, 1–11. 10.1155/2018/7412304 PMC6312624 30662469

[B35] Huang, S S. Leung V. Y. Long D. Chan D. Lu W. W. Cheung K. M. (2013). Coupling of small leucine-rich proteoglycans to hypoxic survival of a progenitor cell-like subpopulation in Rhesus Macaque intervertebral disc. Biomaterials 34 (28), 6548–6558. 10.1016/j.biomaterials.2013.05.027 23764115

[B36] Huang S. Tam V. Cheung K. M. Long D. Lv M. Wang T. (2011). Stem cell-based approaches for intervertebral disc regeneration. Curr. Stem Cell Res. Ther. 6 (4), 317–326. 10.2174/157488811797904335 21190533

[B37] Huang, Y. C Y. C. Leung V. Y. L. Lu W. W. Luk K. D. K. (2013). The effects of microenvironment in mesenchymal stem cell-based regeneration of intervertebral disc. Spine J. 13 (3), 352–362. 10.1016/j.spinee.2012.12.005 23340343

[B38] Huang Y. C. Urban J. P. G. Luk K. D. K. (2014). OPINION intervertebral disc regeneration: do nutrients lead the way? Nat. Rev. Rheumatol. 10 (9), 561–566. 10.1038/nrrheum.2014.91 24914695

[B39] Hwang O. K. Noh Y. W. Hong J. T. Lee J. W. (2020). Hypoxia pretreatment promotes chondrocyte differentiation of human adipose-derived stem cells via vascular endothelial growth factor. Tissue Eng. Regen. Med. 17 (3), 335–350. 10.1007/s13770-020-00265-5 32451775 PMC7260353

[B40] Jacobsen T. D. Hernandez P. A. Chahine N. O. (2021). Inhibition of toll-like receptor 4 protects against inflammation-induced mechanobiological alterations to intervertebral disc cells. Eur. Cell. Mat. 41, 576–591. 10.22203/eCM.v041a37 PMC8329983 34013512

[B41] Jia H. Lin X. Wang D. Wang J. Shang Q. He X. (2022). Injectable hydrogel with nucleus pulposus-matched viscoelastic property prevents intervertebral disc degeneration. J. Orthop. Transl. 33, 162–173. 10.1016/j.jot.2022.03.006 PMC8980713 35415072

[B42] Johnson W. E. Wootton A. El Haj A. Eisenstein S. M. Curtis A. S. Roberts S. (2006). Topographical guidance of intervertebral disc cell growth *in vitro*: towards the development of tissue repair strategies for the anulus fibrosus. Eur. Spine J. 15, S389–S396. 10.1007/s00586-006-0125-9 16688474 PMC2335384

[B43] Kim H. Hong J. Y. Lee J. Jeon W. J. Ha I. H. (2021). IL-1β promotes disc degeneration and inflammation through direct injection of intervertebral disc in a rat lumbar disc herniation model. Spine J. 21 (6), 1031–1041. 10.1016/j.spinee.2021.01.014 33460811

[B44] Kim K. W. Lim T. H. Kim J. G. Jeong S. T. Masuda K. An H. S. (2003). The origin of chondrocytes in the nucleus pulposus and histologic findings associated with the transition of a notochordal nucleus pulposus to a fibrocartilaginous nucleus pulposus in intact rabbit intervertebral discs. Spine (Phila Pa 1976) 28 (10), 982–990. 10.1097/01.BRS.0000061986.03886.4F 12768135

[B45] Kong L. C. Li H. A. Kang Q. L. Li G. (2020). An update to the advances in understanding distraction histogenesis: From biological mechanisms to novel clinical applications. J. Orthop. Transl. 25, 3–10. 10.1016/j.jot.2020.09.003

[B46] Kumar H. Ha D. H. Lee E. J. Park J. H. Shim J. H. Ahn T. K. (2017). Safety and tolerability of intradiscal implantation of combined autologous adipose-derived mesenchymal stem cells and hyaluronic acid in patients with chronic discogenic low back pain: 1-year follow-up of a phase I study. Stem Cell Res. Ther. 8, 262. ARTN 262. 10.1186/s13287-017-0710-3 29141662 PMC5688755

[B47] Lama P. Le Maitre C. L. Harding I. J. Dolan P. Adams M. A. (2018). Nerves and blood vessels in degenerated intervertebral discs are confined to physically disrupted tissue. J. Anat. 233 (1), 86–97. 10.1111/joa.12817 29708266 PMC5987834

[B48] Lang G. Obri K. Saravi B. Boccaccini A. R. Fruh A. Seidenstucker M. (2021). Architecture-promoted biomechanical performance-tuning of tissue-engineered constructs for biological intervertebral disc replacement. Mater. (Basel) 14 (10), 2692. 10.3390/ma14102692 PMC8160686 34065565

[B49] Le Maitre C. L. Freemont A. J. Hoyland J. A. (2004). Localization of degradative enzymes and their inhibitors in the degenerate human intervertebral disc. J. Pathol. 204 (1), 47–54. 10.1002/path.1608 15307137

[B50] Lee K. K. Teo E. C. (2004). Poroelastic analysis of lumbar spinal stability in combined compression and anterior shear. J. Spinal Disord. Tech. 17 (5), 429–438. 10.1097/01.bsd.0000109835.59382.9c 15385884

[B51] Li H. Liang C. Tao Y. Zhou X. Li F. Chen G. (2012). Acidic pH conditions mimicking degenerative intervertebral discs impair the survival and biological behavior of human adipose-derived mesenchymal stem cells. Exp. Biol. Med. (Maywood). 237 (7), 845–852. 10.1258/ebm.2012.012009 22829705

[B52] Li Y. Y. Diao H. J. Chik T. K. Chow C. T. An X. M. Leung V. (2014). Delivering mesenchymal stem cells in collagen microsphere carriers to rabbit degenerative disc: reduced risk of osteophyte formation. Tissue Eng. Part A 20 (9-10), 1379–1391. 10.1089/ten.TEA.2013.0498 24372278 PMC4011461

[B53] Liang H. Chen S. Huang D. Deng X. Ma K. Shao Z. (2018). Effect of compression loading on human nucleus pulposus-derived mesenchymal stem cells. Stem Cells Int. 2018, 1–10. 10.1155/2018/1481243 PMC6196892 30402107

[B54] Liang L. Li X. Li D. Jiang W. Wang H. Chen J. (2017). The characteristics of stem cells in human degenerative intervertebral disc. Med. Baltim. 96 (25), e7178. 10.1097/MD.0000000000007178 PMC5484206 28640098

[B55] Lin H. A. Gupta M. S. Varma D. M. Gilchrist M. L. Nicoll S. B. (2016). Lower crosslinking density enhances functional nucleus pulposus-like matrix elaboration by human mesenchymal stem cells in carboxymethylcellulose hydrogels. J. Biomed. Mat. Res. A 104 (1), 165–177. 10.1002/jbm.a.35552 26256108

[B56] Liu C. Guo Q. Li J. Wang S. Wang Y. Li B. (2014). Identification of rabbit annulus fibrosus-derived stem cells. PLoS One 9 (9), e108239. 10.1371/journal.pone.0108239 25259600 PMC4178129

[B57] Liu J. Tao H. Wang H. Dong F. Zhang R. Li J. (2017). Biological behavior of human nucleus pulposus mesenchymal stem cells in response to changes in the acidic environment during intervertebral disc degeneration. Stem Cells Dev. 26 (12), 901–911. 10.1089/scd.2016.0314 28298159

[B58] Liu L. T. Huang B. Li C. Q. Zhuang Y. Wang J. Zhou Y. (2011). Characteristics of stem cells derived from the degenerated human intervertebral disc cartilage endplate. PLoS One 6 (10), e26285. 10.1371/journal.pone.0026285 22028847 PMC3196539

[B59] Lou J. Stowers R. Nam S. Xia Y. Chaudhuri O. (2018). Stress relaxing hyaluronic acid-collagen hydrogels promote cell spreading, fiber remodeling, and focal adhesion formation in 3D cell culture. Biomaterials 154, 213–222. 10.1016/j.biomaterials.2017.11.004 29132046

[B60] Luo L. Gong J. Wang Z. Liu Y. Cao J. Qin J. (2022). Injectable cartilage matrix hydrogel loaded with cartilage endplate stem cells engineered to release exosomes for non-invasive treatment of intervertebral disc degeneration. Bioact. Mat. 15, 29–43. 10.1016/j.bioactmat.2021.12.007 PMC8940768 35386360

[B61] Luo L. Jian X. Sun H. Qin J. Wang Y. Zhang J. (2021). Cartilage endplate stem cells inhibit intervertebral disc degeneration by releasing exosomes to nucleus pulposus cells to activate Akt/autophagy. Stem Cells 39 (4), 467–481. 10.1002/stem.3322 33459443 PMC8048856

[B62] Lyu F. J. Cheung K. M. Zheng Z. Wang H. Sakai D. Leung V. Y. (2019). IVD progenitor cells: a new horizon for understanding disc homeostasis and repair. Nat. Rev. Rheumatol. 15 (2), 102–112. 10.1038/s41584-018-0154-x 30643232

[B63] Mahmoud M. Kokozidou M. Auffarth A. Schulze-Tanzil G. (2020). The relationship between diabetes mellitus type II and intervertebral disc degeneration in diabetic rodent models: a systematic and comprehensive review. Cells 9 (10), 2208. 10.3390/cells9102208 PMC7600368 33003542

[B64] Marimuthu C. Pushpa Rani V. (2021). Elucidating the role of cell-mediated inflammatory cytokines on allogeneic mouse-derived nucleus pulposus mesenchymal stem cells. J. Food Biochem. 45 (4), e13681. 10.1111/jfbc.13681 33694170

[B65] Meisel H. J. Siodla V. Ganey T. Minkus Y. Hutton W. C. Alasevic O. J. (2007). Clinical experience in cell-based therapeutics: disc chondrocyte transplantation. Biomol. Eng. 24 (1), 5–21. 10.1016/j.bioeng.2006.07.002 16963315

[B66] Meng X. Zhuang L. Wang J. Liu Z. Wang Y. Xiao D. (2018). Hypoxia-inducible factor (HIF)-1alpha knockout accelerates intervertebral disc degeneration in mice. Int. J. Clin. Exp. Pathol. 11 (2), 548 31938140 PMC6957989

[B67] Merceron C. Mangiavini L. Robling A. Wilson T. L. Giaccia A. J. Shapiro I. M. (2014). Loss of HIF-1α in the notochord results in cell death and complete disappearance of the nucleus pulposus. PLoS One 9 (10), e110768. 10.1371/journal.pone.0110768 25338007 PMC4206488

[B68] Navaro Y. Bleich-Kimelman N. Hazanov L. Mironi-Harpaz I. Shachaf Y. Garty S. (2015). Matrix stiffness determines the fate of nucleus pulposus-derived stem cells. Biomaterials 49, 68–76. 10.1016/j.biomaterials.2015.01.021 25725556

[B69] Navone S. E. Marfia G. Canzi L. Ciusani E. Canazza A. Visintini S. (2012). Expression of neural and neurotrophic markers in nucleus pulposus cells isolated from degenerated intervertebral disc. J. Orthop. Res. 30 (9), 1470–1477. 10.1002/jor.22098 22374745

[B70] Nerurkar N. L. Sen S. Baker B. M. Elliott D. M. Mauck R. L. (2011). Dynamic culture enhances stem cell infiltration and modulates extracellular matrix production on aligned electrospun nanofibrous scaffolds. Acta Biomater. 7 (2), 485–491. 10.1016/j.actbio.2010.08.011 20728589 PMC2994961

[B71] Noriega D. C. Ardura F. Hernandez-Ramajo R. Martin-Ferrero M. A. Sanchez-Lite I. Toribio B. (2017). Intervertebral disc repair by allogeneic mesenchymal bone marrow cells: a randomized controlled trial. Transplantation 101 (8), 1945–1951. 10.1097/Tp.0000000000001484 27661661

[B72] Oehme D. Goldschlager T. Rosenfeld J. V. Ghosh P. Jenkin G. (2015). The role of stem cell therapies in degenerative lumbar spine disease: a review. Neurosurg. Rev. 38 (3), 429–445. 10.1007/s10143-015-0621-7 25749802

[B73] Orozco L. Soler R. Morera C. Alberca M. Sanchez A. Garcia-Sancho J. (2011). Intervertebral disc repair by autologous mesenchymal bone marrow cells: a pilot study. Transplantation 92 (7), 822–828. 10.1097/TP.0b013e3182298a15 21792091

[B74] Peck S. H. Bendigo J. R. Tobias J. W. Dodge G. R. Malhotra N. R. Mauck R. L. (2021). Hypoxic preconditioning enhances bone marrow-derived mesenchymal stem cell survival in a low oxygen and nutrient-limited 3D microenvironment. Cartilage 12 (4), 512–525. 10.1177/1947603519841675 30971109 PMC8461160

[B75] Peng Y. Qing X. Shu H. Tian S. Yang W. Chen S. (2021). Proper animal experimental designs for preclinical research of biomaterials for intervertebral disc regeneration. Biomater. Transl. 2 (2), 91–142. 10.12336/biomatertransl.2021.02.003 35836965 PMC9255780

[B76] Penolazzi L. Pozzobon M. Bergamin L. S. D'Agostino S. Francescato R. Bonaccorsi G. (2020). Extracellular matrix from decellularized wharton's jelly improves the behavior of cells from degenerated intervertebral disc. Front. Bioeng. Biotechnol. 8, 262. 10.3389/fbioe.2020.00262 32292779 PMC7118204

[B77] Qi L. Wang R. Shi Q. Yuan M. Jin M. Li D. (2019). Umbilical cord mesenchymal stem cell conditioned medium restored the expression of collagen II and aggrecan in nucleus pulposus mesenchymal stem cells exposed to high glucose. J. Bone Min. Metab. 37 (3), 455–466. 10.1007/s00774-018-0953-9 30187277

[B78] Rasoulian A. Vakili-Tahami F. Smit T. H. (2021). Linear and nonlinear biphasic mechanical properties of goat IVDs under different swelling conditions in confined compression. Ann. Biomed. Eng. 49 (12), 3296–3309. 10.1007/s10439-021-02856-2 34480262

[B79] Risbud M. V. Guttapalli A. Tsai T. T. Lee J. Y. Danielson K. G. Vaccaro A. R. (2007). Evidence for skeletal progenitor cells in the degenerate human intervertebral disc. Spine (Phila Pa 1976) 32 (23), 2537–2544. 10.1097/BRS.0b013e318158dea6 17978651

[B80] Rutges J. P. Duit R. A. Kummer J. A. Oner F. C. van Rijen M. H. Verbout A. J. (2010). Hypertrophic differentiation and calcification during intervertebral disc degeneration. Osteoarthr. Cartil. 18 (11), 1487–1495. 10.1016/j.joca.2010.08.006 20723612

[B81] Sakai D. Andersson G. B. (2015). Stem cell therapy for intervertebral disc regeneration: Obstacles and solutions. Nat. Rev. Rheumatol. 11 (4), 243–256. 10.1038/nrrheum.2015.13 25708497

[B82] Sakai D. Nakamura Y. Nakai T. Mishima T. Kato S. Grad S. (2012). Exhaustion of nucleus pulposus progenitor cells with ageing and degeneration of the intervertebral disc. Nat. Commun. 3, 1264. 10.1038/ncomms2226 23232394 PMC3535337

[B83] See E. Y-S. Toh S. L. Goh J. C. (2012). Simulated intervertebral disc-like assembly using bone marrow-derived mesenchymal stem cell sheets and silk scaffolds for annulus fibrosus regeneration. J. Tissue Eng. Regen. Med. 6 (7), 528–535. 10.1002/term.457 21800436

[B84] Shen Q. Zhang L. Chai B. Ma X. (2015). Isolation and characterization of mesenchymal stem-like cells from human nucleus pulposus tissue. Sci. China Life Sci. 58 (5), 509–511. 10.1007/s11427-015-4839-y 25833805

[B85] Silagi E. S. Schipani E. Shapiro I. M. Risbud M. V. (2021). The role of HIF proteins in maintaining the metabolic health of the intervertebral disc. Nat. Rev. Rheumatol. 17 (7), 426–439. 10.1038/s41584-021-00621-2 34083809 PMC10019070

[B86] Stemper B. D. Baisden J. L. Yoganandan N. Shender B. S. Maiman D. J. (2014). Mechanical yield of the lumbar annulus: a possible contributor to instability. J. Neurosurg-Spine 21 (4), 608–613. 10.3171/2014.6.Spine13401 25084030

[B87] Sun C. Lan W. Li B. Zuo R. Xing H. Liu M. (2019). Glucose regulates tissue-specific chondro-osteogenic differentiation of human cartilage endplate stem cells via O-GlcNAcylation of Sox9 and Runx2. Stem Cell Res. Ther. 10 (1), 357. 10.1186/s13287-019-1440-5 31779679 PMC6883626

[B88] Tao Y. Zhou X. Liang C. Li H. Han B. Li F. (2015). TGF-**β**3 and IGF-1 synergy ameliorates nucleus pulposus mesenchymal stem cell differentiation towards the nucleus pulposus cell type through MAPK/ERK signaling. Growth factors. 33 (5-6), 326–336. 10.3109/08977194.2015.1088532 26431359

[B89] Tsai T. L. Nelson B. C. Anderson P. A. Zdeblick T. A. Li W. J. (2014). Intervertebral disc and stem cells cocultured in biomimetic extracellular matrix stimulated by cyclic compression in perfusion bioreactor. Spine J. 14 (9), 2127–2140. 10.1016/j.spinee.2013.11.062 24882152

[B90] Urits I. Capuco A. Sharma M. Kaye A. D. Viswanath O. Cornett E. M. (2019). Stem cell therapies for treatment of discogenic low back pain: a comprehensive review. Curr. Pain Headache Rep. 23 (9), 65. 10.1007/s11916-019-0804-y 31359164

[B91] Vadala G. Mozetic P. Rainer A. Centola M. Loppini M. Trombetta M. (2012). Bioactive electrospun scaffold for annulus fibrosus repair and regeneration. Eur. Spine J. 21, S20–S26. 10.1007/s00586-012-2235-x 22411039 PMC3325390

[B92] Wan Y. Feng G. Shen F. H. Laurencin C. T. Li X. (2008). Biphasic scaffold for annulus fibrosus tissue regeneration. Biomaterials 29 (6), 643–652. 10.1016/j.biomaterials.2007.10.031 17997480

[B93] Wang X. Wenk E. Zhang X. Meinel L. Vunjak-Novakovic G. Kaplan D. L. (2009). Growth factor gradients via microsphere delivery in biopolymer scaffolds for osteochondral tissue engineering. J. Control. Release 134 (2), 81–90. 10.1016/j.jconrel.2008.10.021 19071168 PMC2698962

[B94] Wang Y. X. J. Kaplar Z. Deng M. Leung J. C. S. (2017). Lumbar degenerative spondylolisthesis epidemiology: a systematic review with a focus on gender-specific and age-specific prevalence. J. Orthop. Transl. 11, 39–52. 10.1016/j.jot.2016.11.001 PMC5866399 29662768

[B95] Wang Z. Cui M. Qu Y. He R. Wu W. Lin H. (2020). Hypoxia protects rat bone marrow mesenchymal stem cells against compression-induced apoptosis in the degenerative disc microenvironment through activation of the HIF-1α/YAP signaling pathway. Stem Cells Dev. 29 (20), 1309–1319. 10.1089/scd.2020.0061 32799744

[B96] Wangler S. Peroglio M. Menzel U. Benneker L. M. Haglund L. Sakai D. (2019). Mesenchymal stem cell homing into intervertebral discs enhances the tie2-positive progenitor cell population, prevents cell death, and induces a proliferative response. Spine (Phila Pa 1976) 44 (23), 1613–1622. 10.1097/BRS.0000000000003150 31730570 PMC6867676

[B97] Wei Q. Wang S. Han F. Wang H. Zhang W. Yu Q. (2021). Cellular modulation by the mechanical cues from biomaterials for tissue engineering. Biomater. Transl. 2 (4), 323–342. 10.12336/biomatertransl.2021.04.001 35837415 PMC9255801

[B98] Wu D. Wu X. Wu J. Tam L. S. Gu J. (2020). Fri0552 Global, regional, and national burden of low back pain, 1990-2019: a systematic analysis for the global burden of disease study 2019. Ann. Rheum. Dis. 79, 877.1–878. 10.1136/annrheumdis-2020-eular.2602

[B99] Xu Z. Zheng J. Zhang Y. Wu H. Sun B. Zhang K. (2021). Increased expression of integrin alpha 6 in nucleus pulposus cells in response to high oxygen tension protects against intervertebral disc degeneration. Oxid. Med. Cell. Longev. 2021, 1–16. 10.1155/2021/8632823 PMC8545551 34707783

[B100] Yang H. Cao C. Wu C. Yuan C. Gu Q. Shi Q. (2015). TGF-Βl suppresses inflammation in cell therapy for intervertebral disc degeneration. Sci. Rep. 5, 13254. 10.1038/srep13254 26289964 PMC4542522

[B101] Yang H. Wu J. Liu J. Ebraheim M. Castillo S. Liu X. (2010). Transplanted mesenchymal stem cells with pure fibrinous gelatin-transforming growth factor-β1 decrease rabbit intervertebral disc degeneration. Spine J. 10 (9), 802–810. 10.1016/j.spinee.2010.06.019 20655810

[B102] Yao J. Turteltaub S. R. Ducheyne P. (2006). A three-dimensional nonlinear finite element analysis of the mechanical behavior of tissue engineered intervertebral discs under complex loads. Biomaterials 27 (3), 377–387. 10.1016/j.biomaterials.2005.06.036 16168476

[B103] Yao Y. Deng Q. Sun C. Song W. Liu H. Zhou Y. (2017a). A genome-wide analysis of the gene expression profiles and alternative splicing events during the hypoxia-regulated osteogenic differentiation of human cartilage endplate-derived stem cells. Mol. Med. Rep. 16 (2), 1991–2001. 10.3892/mmr.2017.6846 28656244 PMC5562021

[B104] Yao Y. Shang J. Song W. Deng Q. Liu H. Zhou Y. (2016). Global profiling of the gene expression and alternative splicing events during hypoxia-regulated chondrogenic differentiation in human cartilage endplate-derived stem cells. Genomics 107 (5), 170–177. 10.1016/j.ygeno.2016.03.003 26996146

[B105] Yao Y. Song W. Deng Q. Zhang H. Wang J. Liu H. (2017b). General regulatory effects of hypoxia on human cartilage endplatederived stem cells: a genomewide analysis of differential gene expression and alternative splicing events. Mol. Med. Rep. 16 (3), 3001–3009. 10.3892/mmr.2017.6907 28677762

[B106] Yin X. Motorwala A. Vesvoranan O. Levene H. B. Gu W. Huang C. Y. (2020). Effects of glucose deprivation on ATP and proteoglycan production of intervertebral disc cells under hypoxia. Sci. Rep. 10 (1), 8899. 10.1038/s41598-020-65691-w 32483367 PMC7264337

[B107] Yoshikawa T. Ueda Y. Miyazaki K. Koizumi M. Takakura Y. (2010). Disc regeneration therapy using marrow mesenchymal cell transplantation a report of two case studies. Spine 35 (11), E475–E480. 10.1097/BRS.0b013e3181cd2cf4 20421856

[B108] Yuan C. Pu L. He Z. Wang J. (2018). BNIP3/Bcl-2-mediated apoptosis induced by cyclic tensile stretch in human cartilage endplate-derived stem cells. Exp. Ther. Med. 15 (1), 235–241. 10.3892/etm.2017.5372 29375685 PMC5763692

[B109] Yue Y. Wang X. Han J. Yu L. Chen J. Wu Q. (2019). Effects of nanocellulose on sodium alginate/polyacrylamide hydrogel: mechanical properties and adsorption-desorption capacities. Carbohydr. Polym. 206, 289–301. 10.1016/j.carbpol.2018.10.105 30553324

[B110] Zehra U. Tryfonidou M. Iatridis J. C. Illien-Junger S. Mwale F. Samartzis D. (2022). Mechanisms and clinical implications of intervertebral disc calcification. Nat. Rev. Rheumatol. 18 (6), 352–362. 10.1038/s41584-022-00783-7 35534553 PMC9210932

[B111] Zhang W. Chu G. Wang H. Chen S. Li B. Han F. (2020a). Effects of matrix stiffness on the differentiation of multipotent stem cells. Curr. Stem Cell Res. Ther. 15 (5), 449–461. 10.2174/1574888X15666200408114632 32268870

[B112] Zhang W. Wang H. Yuan Z. Chu G. Sun H. Yu Z. (2021a). Moderate mechanical stimulation rescues degenerative annulus fibrosus by suppressing caveolin-1 mediated pro-inflammatory signaling pathway. Int. J. Biol. Sci. 17 (5), 1395–1412. 10.7150/ijbs.57774 33867854 PMC8040478

[B113] Zhang Y. Hu Y. Wang W. Guo Z. Yang F. Cai X. (2020b). Current progress in the endogenous repair of intervertebral disk degeneration based on progenitor cells. Front. Bioeng. Biotechnol. 8, 629088. 10.3389/fbioe.2020.629088 33553131 PMC7862573

[B114] Zhang Y. Jiang Y. Zou D. Yuan B. Ke H. Z. Li W. (2021b). Therapeutics for enhancement of spinal fusion: a mini review. J. Orthop. Transl. 31, 73–79. 10.1016/j.jot.2021.11.001 PMC8660701 34934624

[B115] Zhang Y. Wang Y. Zhou X. Wang J. Shi M. Wang J. (2020c). Osmolarity controls the differentiation of adipose-derived stem cells into nucleus pulposus cells via histone demethylase KDM4B. Mol. Cell. Biochem. 472 (1-2), 157–171. 10.1007/s11010-020-03794-8 32594337

[B116] Zhao Y. Qin Y. Wu S. Huang D. Hu H. Zhang X. (2020). Mesenchymal stem cells regulate inflammatory milieu within degenerative nucleus pulposus cells via p38;MAPK pathway. Exp. Ther. Med. 20 (5), 1. 10.3892/etm.2020.9150 32934687 PMC7471866

[B117] Zhou P. Chu G. Yuan Z. Wang H. Zhang W. Mao Y. (2021). Regulation of differentiation of annulus fibrosus-derived stem cells using heterogeneous electrospun fibrous scaffolds. J. Orthop. Transl. 26, 171–180. 10.1016/j.jot.2020.02.003 PMC7773966 33437636

[B118] Zhu C. Li J. Liu C. Zhou P. Yang H. Li B. (2016). Modulation of the gene expression of annulus fibrosus-derived stem cells using poly(ether carbonate urethane)urea scaffolds of tunable elasticity. Acta Biomater. 29, 228–238. 10.1016/j.actbio.2015.09.039 26432437

[B119] Zuo R. Wang Y. Li J. Wu J. Wang W. Li B. (2019). Rapamycin induced autophagy inhibits inflammation-mediated endplate degeneration by enhancing nrf2/keap1 signaling of cartilage endplate stem cells. Stem Cells 37 (6), 828–840. 10.1002/stem.2999 30840341

